# Using Cellular Automata to Simulate Domain Evolution in Proteins

**DOI:** 10.3389/fgene.2020.00515

**Published:** 2020-06-09

**Authors:** Xuan Xiao, Guang-Fu Xue, Biljana Stamatovic, Wang-Ren Qiu

**Affiliations:** ^1^Computer Department, Jing-De-Zhen Ceramic Institute, Jingdezhen, China; ^2^Faculty of Information Systems and Technologies, University of Donja Gorica, Podgorica, Montenegro

**Keywords:** simulation, protein evolution, cellular automaton, multi-domain proteins, protein domain architecture

## Abstract

Proteins play primary roles in important biological processes such as catalysis, physiological functions, and immune system functions. Thus, the research on how proteins evolved has been a nuclear question in the field of evolutionary biology. General models of protein evolution help to determine the baseline expectations for evolution of sequences, and these models have been extensively useful in sequence analysis as well as for the computer simulation of artificial sequence data sets. We have developed a new method of simulating multi-domain protein evolution, including fusions of domains, insertion, and deletion. It has been observed via the simulation test that the success rates achieved by the proposed predictor are remarkably high. For the convenience of the most experimental scientists, a user-friendly web server has been established at http://jci-bioinfo.cn/domainevo, by which users can easily get their desired results without having to go through the detailed mathematics. Through the simulation results of this website, users can predict the evolution trend of the protein domain architecture.

## Introduction

Proteins are the biological macromolecular entities most close-knitly related to organismal functions. Evolution in the sequences of proteins results in the way these proteins function, and protein evolution is a critical component of organismal evolution and a valuable method for generating useful molecules in the laboratory (Leconte et al., 2013). Therefore, the research on protein evolution plays an elementary and central role in computational proteomics. Experimental efforts to understand protein evolution have largely depended on the reconstruction of hypothetic evolutionary intermediates or on experimental evolution over modest numbers of rounds of evolution (Weinreich et al., 2006; Gumulya et al., 2012). Long evolutionary trajectory experiments have met challenges in studying proteins but have been successfully executed only for whole organisms and RNA. Directed evolution has been a powerful technique for generating tailor-made enzymes for a wide range of biocatalytic applications (Zeymer and Hilvert, 2018), but it is both time- and money-consuming to study protein evolution by conducting experiments alone. With the rapid development of computational power, hidden Markov model based on statistics, phylogeny model based on Bayesian statistics, and better prediction method of protein structure, the ability to model evolutionary processes in proteins has improved.

Protein evolution is modeled firstly by considering the amino acid substitution process. Dayhoff et al. (1978) proposed the most influential amino acid substitution model. This simple model supposes that all sites in the protein sequence are independent of each other during protein evolution, and that each site mutation depends on an amino acid replacement matrix. Since then, many protein evolution models based on amino acid substitution matrices have been proposed, such as the JTT model (Jones et al., 1992), the mtREV model (Adachi and Hasegawa, 1996), and the WAG model (Whelan and Goldman, 2001). However, in most cases, the assumption that “the proteins are independent of each other during evolution” is not consistent with the fact that any amino acid residue within the protein interacts with its neighboring amino acids. Yang (1993) has designed an ingenious method that allows variant sites in the amino acid sequence to have variant rates of evolution. This method basically classifies amino acids according to their physicochemical properties, making amino acids with similar properties more likely to be replaced (Yang, 1993). Protein evolution is driven by the sum of variant physiochemical and genetic processes that usually results in strong purifying selection to maintain biochemical functions. However, proteins that are part of systems under arms race dynamics often evolve at unparalleled rates that can produce atypical biochemical properties (Wilburn et al., 2018).

Phylogenetic methods have been widely used to analyze the evolutionary history of protein sequences. The simulation of sequences is one means of investigating phylogenetic hypotheses (Tuffery, 2002). There are many more powerful bioinformatics tools for such simulations (Bakan et al., 2014). Sirakoulis et al. (2003) used a cellular automaton (CA) model for the study of DNA sequence evolution where DNA is modeled as a one-dimensional (1D) CA with four states per cell which correspond to four DNA bases. Moreover, they have developed genetic algorithms in order to determine the rules of CA evolution that simulate the DNA evolution course. Simulation models for protein evolution based on CA have lagged far behind models of DNA evolution because proteins are composed of 20 amino acids, while DNA is composed of only four nucleotides. Many authors developed different simulations of protein evolution, but few of them are operated by non-expert users. They are either very specific for certain needs or distributed as non-interactive command-line programs or require a complex preparation of the input data. This precludes these techniques being used by most molecular biologists.

Proteins are composed of domains, recurrent protein fragments with distinct structure and function, and proteins can be classed as single-domain proteins or multi-domain proteins (Chothia, 1992; Riley and Labedan, 1997). The structural domain databases SCOP and CATH were gathered based on identifying recurring elements in experimentally determined protein three-dimensional (3D) structures (Dawson et al., 2016; Chandonia et al., 2017). In Pfam databases, conserved regains are identified from sequence analysis and background knowledge to make multiple sequence alignments (El-Gebali et al., 2019). Domain definitions form different databased only partially overlap; however, the choice of database appears to have little effect on modeling the evolution of protein domain architectures (Apic et al., 2001). Domain architecture generally refers to the domains in a protein and their order, reported in N- to C-terminal direction along the amino acid chain. The mechanisms for domain architecture change can be classed into new domain, fission, and fusion (Fong et al., 2007). The multi-domain architectures usually evolve from existing architectures because few multi-domain architectures contain all new domain. Fusion class would be partitioned into three sub-cases as fusion of new domains, fusion of parent architectures, and fusion of parent architecture and new domain. Snel et al. (2000) summarized that domain fusions are more common that domain fissions, and the result was subsequently supported by a larger study by Kummerfeld and Teichmann (2005). Buljan and Bateman observed that domain architecture changes primarily take place at the protein termini and it can be explained from that terminal changes to the architecture are less likely to disturb overall protein structure (Buljan and Bateman, 2009), and similar results have been found in several other studies (Buljan et al., 2010). Zhang et al. (2012) and Sharma and Pandey (2016) studied the role of gene duplication in plants protein domain architecture evolution. More recently, Wiedenhoeft et al. (2011) used a network construct named as plexus to reconstruct domain architecture history. Stolzer et al. (2015) present another method for domain architecture history inference, made available through the Notung software.

Multi-domain proteins have evolved by insertions or deletions of distinct protein domains. We have a general understanding of the mechanisms of protein domain architecture evolution based on the aforementioned models. Here we introduce a new protein evolution simulation model to simulate the evolution of protein domains by 1D CA. In the model, the HMMER (Prakash et al., 2017) and Pfam databases are united in the process for annotating the protein domains, and it can be easily to simulate the evolution of the domain architecture in the multi-domain protein family. Furthermore, the model may obtain new domain architecture which may be the potential protein evolution.

## Methods And Implementation

### Data Preprocessing

After receiving the protein sequence file P (*P* = {*p*_1_, *p*_2_, *p*_3_, …, *p*_*n*_}, where *p*_1_, *p*_2_, *p*_3_, …, *p*_*n*_ represent the protein sequence in the file) in FASTA format, the system annotates the protein domain of each protein sequence *p*_*i*_ based on the HMMER and Pfam databases, and each protein *p*_*i*_ generates a corresponding annotation file *f*_*i*_. By analyzing the annotation file *f*_*i*_, the domain information of protein *p*_*i*_ can be screened out and expressed as a multi-domain sequence:

(1) $$\begin{array}{r} p_{i}=\left\{d_{i,1},d_{i,2},d_{i,3},\ldots ,d_{i,k}\right\} \end{array}$$

where *d*_*i*, 1_, *d*_*i*, 2_, *d*_*i*, 3_, …, *d*_*i, k*_ represent the homologous domain of the protein *p*_*i*_, *k* is the number of domains in protein *p*_*i*_. According to the ACC (the average posterior probability of the aligned target sequence residues) value of each domain, we determine the position of these domains in the protein sequence or the order of domains in the sequence. The protein *p*_i_ is expressed as an ordered multi-domain sequence ${p_{i}}^{\prime }$ based on the context of these domains:

(2) $${p_{i}}^{\prime }={d_{i,1}}^{\prime },{d_{i,2}}^{\prime },{d_{i,3}}^{\prime },\ldots ,{d_{i,k}}^{\prime }$$

The protein sequence file P is expressed as a set of multi-domain sequences as file P′:

(3) $$\text{P}=\left(\begin{array}{c} {{\text{ p}}_{1}}^{\prime }\\ {{\text{p}}_{2}}^{\prime }\\ \begin{array}{l} .\\ .\\ . \end{array}\\ {p_{n}}^{\prime } \end{array}\right)=\left(\begin{array}{l} {{\text{d}}_{1,1}}^{\prime },{{\text{d}}_{2,1}}^{\prime },...,{{\text{d}}_{1,w}}^{\prime }\\ {{\text{d}}_{2,1}}^{\prime },{{\text{d}}_{2,2}}^{\prime },...,{{\text{d}}_{2,u}}^{\prime }\\ \;.\\ \;.\;\\ \;.\\ {{\text{d}}_{n,1}}^{\prime },{{\text{d}}_{n,2}}^{\prime },...,{{\text{d}}_{n,l}}^{\prime } \end{array}\right)$$

where *w* is the number of domains in protein ${p_{1}}^{\prime }$, *u* is the number of domains in protein ${p_{2}}^{\prime }$, and so forth. Therefore, a considerate protein sequence file P in FASTA format is processed by the above methods to form the training data set *P*′ for the proposed evolutionary simulation model.

Inspired by incorporating the dipeptide position-specific propensity into the general pseudo nucleotide composition (Xiao et al., 2016), here we develop a new method to simulate the protein evolution process by domain position-specific propensity.

If *g* domain classes appeared in the training data set *P*′, we added two termination symbols “*X*-start” and “*X*-end” in the front and the back of the ${p_{i}}^{\prime }$, there are (*g*^2^+*g* × 2) couple: *X*-*startD*_1_, *X*-*startD*_2_,…, *D*_1_*D*_1_ (where domain D_1_ and domain D_1_ are connected together), *D*_1_*D*_2_, *D*_1_*D*_3_,…, *D*_*g*_*D*_*g*_, *D*_1_*X*. -*ending*,…, *D*_*g*_*X*-*ending*. Thus, for the training data set *P*′, its profile (or detailed information) of mmarized by the following two matrices:

(4) $$\begin{array}{r} F2B=\left[\begin{array}{cccccc} X\text{–}start\;D_{1} & D_{1}D_{1} & D_{1}D_{2} & \cdots & D_{1}D_{g} & D_{1}X\text{–}end\\ X\text{–}start\;D_{2} & D_{2}D_{1} & D_{2}D_{2} & \cdots & D_{2}D_{g} & D_{2}X\text{–}end\\ \vdots & \vdots & \vdots & \cdots & \vdots & \vdots \\ X\text{–}start\;D_{g} & D_{g}D_{1} & D_{g}D_{2} & \cdots & D_{g}D_{g} & D_{g}X\text{–}end \end{array}\right] \end{array}$$

(5) $$B2F=\left[\begin{array}{llllll} X-start{D_{1}}^{\prime } & {D_{1}}^{\prime }{D_{1}}^{\prime } & {D_{1}}^{\prime }{D_{2}}^{\prime } & ... & {D_{1}}^{\prime }{D_{g}}^{\prime } & {D_{1}}^{\prime }X-end\\ X-start{D_{2}}^{\prime } & {D_{2}}^{\prime }{D_{1}}^{\prime } & {D_{2}}^{\prime }{D_{2}}^{\prime } & ... & {D_{2}}^{\prime }{D_{g}}^{\prime } & {D_{2}}^{\prime }X-end\\ \begin{array}{l} \;.\\ \;.\\ \;. \end{array} & \begin{array}{l} \;.\\ \;.\\ \;. \end{array} & \begin{array}{l} \;.\\ \;.\\ \;. \end{array} & ... & \begin{array}{l} \;.\\ \;.\\ \;. \end{array} & \begin{array}{l} \;.\\ \;.\\ \;. \end{array}\\ X-start{D_{g}}^{\prime } & {D_{g}}^{\prime }{D_{1}}^{\prime } & {D_{g}}^{\prime }{D_{2}}^{\prime } & ... & {D_{g}}^{\prime }{D_{g}}^{\prime } & {D_{g}}^{\prime }X-end \end{array}\right]$$

where *F*2*B*(from front to back) is matrix of evolution prior probability from front to back and *B*2*F*(from back to front) is the matrix of evolution prior probability from back to front. In the matrix *F*2*B*, *D*_*i*_*D*_*j*_ (1 ≤ *i* ≤ *g*, 1 ≤ *j* ≤ *g*) is the occurrence frequency of domain*D*_*i*_ attached to *D*_*j*_, and *D*_*j*_ behind *D*_*i*_. In the matrix *B*2*F*, ${D_{i}}^{\prime }$${D_{i}}^{\prime }$ is the occurrence frequency of domain *D*_*i*_ attached to *D*_*j*_, and *D*_*i*_ is followed by *D*_*j*_.

### Simulation Model of Domain Evolution in Proteins Based on Cellular Automaton

**Figure 1:**
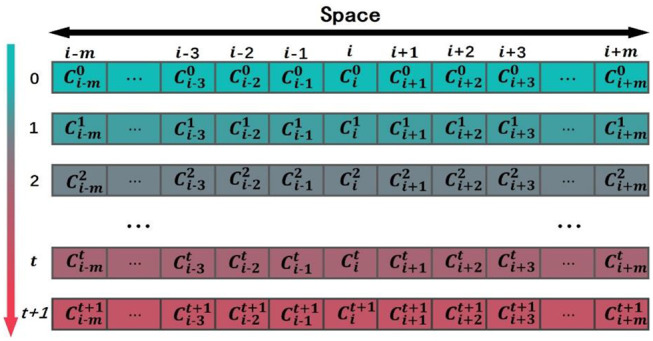
The evolution of a one-dimensional cellular automaton (CA).

Let us give a brief introduction to CA. A CA is a dynamical system in which space, time, and the states are discrete. Each cell, defined by a point in a regular spatial lattice, can be any one of a finite number of states that are updated according to a local rule (Schwartz et al., 1967; Chopard and Droz, 1998). In 1D CA, the lattice consists of identical cells, *i*-*m*, …, *i*-3, *i*-2, *i*-1, *i, i*+1, *i*+2, *i*+3, …, *i*+*m*, and the corresponding states of these cells are *C*_*i*−*m*_, ⋯ , *C*_*i*−2_, *C*_*i*−1_, *C*_*i*_, *C*_*i*+1_, *C*_*i*+2_, ⋯ , *C*_*i*+*m*_. The symbol *i* is the center of initial sequence with length equals to 2*m*+1. The state of the *ith* cell takes value from a predefined discrete set: *C*_*i*_∈{*c*_1_, *c*_2_, ⋯ , *c*_*Q*_}, where *c*_1_, *c*_2_, ⋯ , *c*_*Q*_ are the elements of the set. The CA evolves in discrete time steps, and its evolution is manifested by the change of its cell states with time. The state of each cell is affected by the states of its neighboring cells. The neighborhood is defined as N(*i*, r) = {*C*_*i*−*r*_, ⋯ , *C*_*i*−1_, *C*_*i*_, *C*_*i*+1_, ⋯ , *C*_*i*+*r*_}, where *r* is the size of the neighborhood. If *r* = 1, the neighborhood of the *ith* cell consists of the same cell and its left and right immediate neighbors N(*i*, 1) = {*C*_*i*−1_, *C*_*i*_, *C*_*i*+1_}. The state of the *ith* cell at time step *t*+1 is affected by the states of its neighbors at the previous time step *t*, $C_{i}^{t+1}=F\left(C_{i-r}^{t},\cdots \;,C_{i-1}^{t},C_{i}^{t},C_{i+1}^{t},\cdots \;,C_{i+r}^{t}\right)$. *F* is the CA evolution rule (Sirakoulis et al., 2003). Figure 1 shows the evolution of a 1D CA. the horizontal axis is space, and the vertical axis is time. Each column represents the state of cell at various time steps.

**Figure 2:**
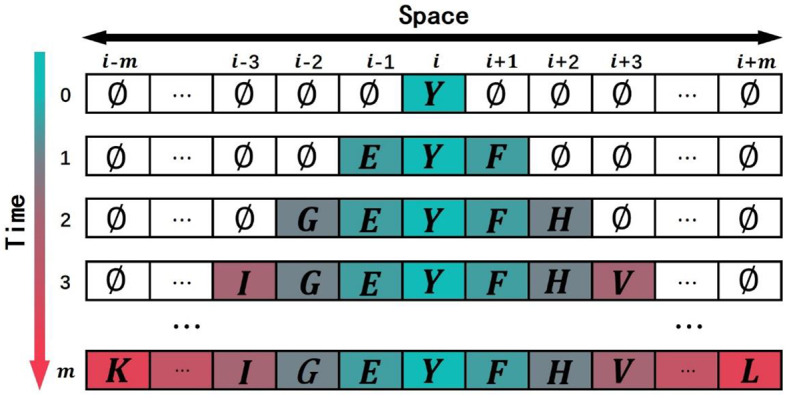
Schematic drawing to show the initial state of one-dimensional cellular automaton (CA) and the course of evolution. Each site of this lattice is called cell. The value of domain is the state of the cell.

In this study, the non-uniform 1D CA was used to simulate the domain evolution in proteins. The square arrays are a very basic data structure in computers, and it was rational to use a square lattice in our model. If protein p_q_ in the training data set P′ has the most domains, and the number of domains is *m*, then the spatial dimension of the 1D CA in the model is 1 × (2*m*+1) and the time step in CA evolution is set as *m*. The model was a (*g*+3)-state model in which each cell in the lattice was one of the following (*g*+3) states: (1) *g* domain classes appeared in the training data set P′; (2) evolution termination symbols “*X*-start”; (3) evolution termination symbols “*X*-*end*”; (4) an empty state “∅″. The state of the cell at time *t*can be expressed as $C_{j}^{t}$. The upper index in the state symbol denotes the time step, and the lower index denotes the cell *j*. When the CA is initialized (*t* = 0), the state of the cell $C_{i}^{0}$ in the middle of the CA is set to the ancestral domain *Y*, and the state of the other cells is set to the empty state ∅, as shown in Figure 2. The state $C_{j}^{t+1}$ of the cell at time *t*+1 is determined by the state $C_{i}^{t}$ of the cell at time *t* and the state $C_{j-1}^{t}$and $C_{j+1}^{t}$ of its neighbor cells at time *t*.

**Figure 3:**
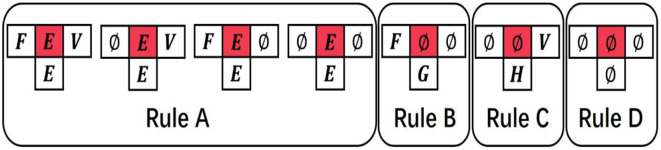
The evolution of cellular automaton (CA). The state of each cell is affected by the states of its neighboring cells.

The evolution rules of the proposed CA model can be expressed as follows (Figure 3):

- Rule A: ***Inheritance***. If the state of cell $C_{j}^{t}$ is domain *E*(*E*≠∅), it means that the cell has evolved into domain *E*, so this cell $C_{j}^{t}$ will inherit the domain at the next time, which means that the state of $C_{j}^{t+1}$ is *E*.
- Rule B: ***Rules of evolution from front to back***. If the state of cells $C_{j}^{t}$ and $C_{j+1}^{t}$ is the empty domain ∅, and the state of cell $C_{j-1}^{t}$ is domain *F*(*F*≠∅), the state $C_{j}^{t+1}$ will be obtained by the Roulette wheel selection algorithm. According to the matrix *F*2*B* (from front to back evolution prior probability matrix), we know that the probability of all domains to appear behind domain *F*. It is not true that the domain of the biggest probability certainly appears behind domain *F* in natural evolution course. Hence, we determined the state $C_{j}^{t+1}$ into one domain form all domains that appear behind domain *F* probability is not zero based on Roulette wheel selection algorithm. The probability of selecting a domain *G* is the same as the probability of domain *F* appearing after domain *G* in matrix *F*2*B*.
- Rule C: ***Rules of evolution from back to front***. If the state of cells $C_{j-1}^{t}$ and $C_{j}^{t}$ is the empty state ∅, and the state of cell $C_{j+1}^{t}$ is domain *V*(*V*≠∅), then the state $C_{j}^{t+1}$ will be obtained by the Roulette wheel selection algorithm. The selected course is similar to Rule B except that the matrix *B*2*F* (from back to front evolution prior probability matrix) was used instead of the matrix *F*2*B*. The probability of selecting a domain *H* being selected is the same as the probability of domain *H* appearing before domain *V* in matrix B2F.
- Rule D: ***Return inanimateness***. If the states of cells $C_{j-1}^{t}$, $C_{j}^{t}$, and $C_{j+1}^{t}$ are all equal to the empty state ∅, the state $C_{j}^{t+1}$ will remain the empty state ∅.

Proteins in a family descend from a common ancestor and have similar 3D structures, functions, and sequence similarity. Thus, the model assumes that all of the evolved proteins contain ancestral domains *Y*(*Y*∈{*D*_1_, *D*_2_, *D*_3_, …, *D*_*g*_, *X*-*start, X*-*end*}), and where the common domains in the P′ are considered to be the hypothetical ancestral domain. By running the model once, a new protein will be simulated, and the evolved protein is represented by an ordered sequence of multi-domains.

**Figure 4:**
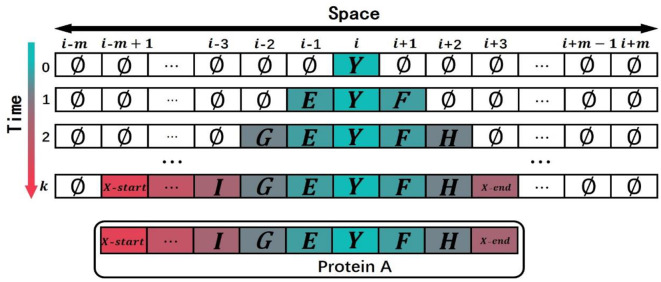
An example to show how the model simulated the domain evolution. The model evolved the termination symbols *X*-start and *X*-end in advance at time *k*, so the protein stopped the evolution at time *k*.

As shown in Figure 4, when the CA is initialized (*t* = 0), the state of the cell $C_{i}^{0}$ in the middle of the CA is set to the ancestral domain *Y*, and the state of the other cells is set to the empty state ∅.

When *t* = 1, the state of $C_{i}^{1}$ is determined by the state of $C_{i-1}^{0},C_{i}^{0}$ and $C_{i+1}^{0}$. Since the state of $C_{i}^{0}$ is the domain *Y*(*Y*≠∅), then $C_{i}^{1}$ will be domain *Y* according to rule A inherit the state of $C_{i}^{0}$; the state of $C_{i-1}^{1}$ is determined by the state of $C_{i-2}^{0},C_{i-1}^{0}$ and $C_{i}^{0}$. Because the state of $C_{i-1}^{0}$ is the empty domain ∅ and the state of $C_{i}^{0}$ is the domain *Y*, the state of $C_{i-1}^{1}$ should be selected by the roulette method according to rule C and the matrix *B*2*F* to obtain the domain *E*. The state of $C_{i+1}^{1}$ is determined by the state of $C_{i}^{0},C_{i+1}^{0}$ and $C_{i+2}^{0}$ with the reason that the state of $C_{i}^{0}$ is the domain *Y* and the state of $C_{i+1}^{0}$ is the domain ∅. According to rule *B*, the state of $C_{i+1}^{1}$should be selected by the roulette method on the basis of the matrix *F*2*B* to obtain the domain *F* after the domain *Y*. Keep the rule *D* in mind, the state of the other cells is the empty domain ∅ because the state of the other cells at the previous moment and the states of their neighbors are the empty domain ∅.

When *t* = 2, the states of $C_{i-1}^{2},C_{i}^{2}$, and $C_{i+1}^{2}$ are determined by the states of $C_{i-1}^{1},C_{i}^{1},C_{i+1}^{1}$and their neighbors with the reason that the states of $C_{i-1}^{1},C_{i}^{1}$ and $C_{i+1}^{1}$ are not domain ∅. According to the rule A, $C_{i-1}^{2},C_{i}^{2},$ and $C_{i+1}^{2}$ will inherit the states, respectively, $C_{i-1}^{1},C_{i}^{1},$ and $C_{i+1}^{1}$ with domains *E*, *Y* and domain *F*; the state of $C_{i-2}^{2}$ is determined by the state of $C_{i-3}^{1},C_{i-2}^{1}$, and $C_{i-1}^{1}$ at the previous time. Because the states of $C_{i-3}^{1}$ and $C_{i-2}^{1}$ are the domain ∅ and the state of $C_{i-1}^{1}$ is the domain *E*, according to rule C, the state of $C_{i-2}^{2}$ will be selected by the roulette method according to the matrix *B*2*F* to obtain the domain *G* in front of the domain *E*; the state of $C_{i+2}^{2}$ is determined by the state of $C_{i+1}^{1},C_{i+2}^{1}$, and $C_{i+3}^{1}$. Because the states of $C_{i+2}^{1}$ and $C_{i+3}^{1}$ are the domain ∅ and the state of $C_{i+1}^{1}$ is the domain *F*, the state of $C_{i+2}^{2}$will be selected by the roulette method according to rule B and the matrix *F*2*B* to obtain the domain *H* after the domain *F*. According to rule D, the state of the other cells is the domain ∅ on the basis of that the state of the other cells and the states of their neighbors are the domain ∅.

When *t* = *k*(*k*<*m*), the cells with the state of termination symbols *X*-start and *X*-*end* are evolved in the cell space, and the simulated evolution of the protein comes to an end. The state of the cell space at time *k* is taken as an ordered sequence of multi-domain, removing the cells with the empty state ∅. The simulated multi-domain sequence of protein A is expressed as:

(6) $$\begin{array}{r} \left\{X\text{–}start\;,\;...\;,I\;,G\;,E\;,Y\;,F\;,H\;,\;...\;,X\text{–}end\;\right\}\\ \left(E\;,F\;,G\;,H\;,I\;,Y\;\in \left\{D_{1},D_{2},D_{3},\ldots ,D_{g}\right\}\right) \end{array}$$

**Figure 5:**
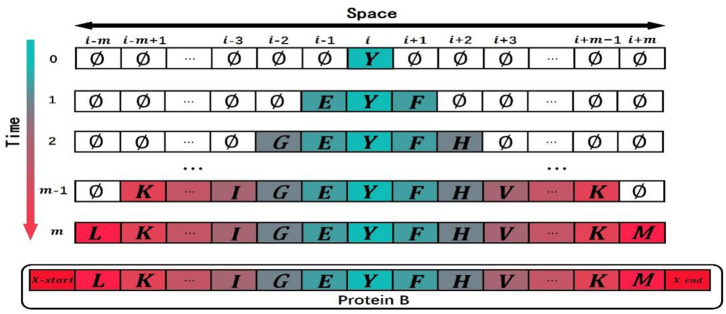
An example to show how to put a termination to domain evolution. When the model is at time *m*, the termination symbols *X*-start and *X*-end have not yet evolved. Since the maximum time step of the cellular automaton is *m*, the protein stops evolution at time *m*.

As shown in Figure 5, when *t* = *m*, if termination symbols *X*-start and *X*-*end* have not evolved in the cell space, the simulated evolution of the protein also comes to an end. The state of the cell space at time *m* is taken as an ordered sequence of multi-domain, removing the cells with the empty state ∅ and adding two termination symbols to the left and right ends of the cell space. The simulated multi-domain sequence of protein B is expressed as:

(7) $$\begin{array}{r} \left\{X\text{–}start\;,L,K,\;...,I,G,E,Y,F,H,V,\;...,K,M,X\text{–}end\;\right\}\\ \left(E,F,G,H,I,J,K,L,M,V,Y\;\in \left\{D_{1},D_{2},D_{3},\ldots ,D_{g}\right\}\right) \end{array}$$

## User Interface

**Figure 6:**
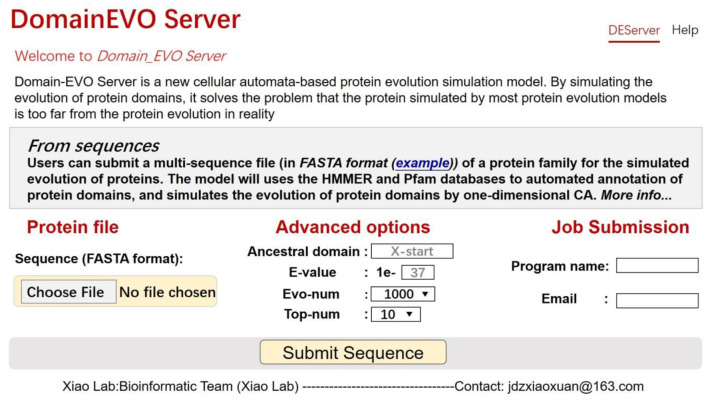
A semi-screenshot to show the top page of the cellular automaton (CA) model web server at http://jci-bioinfo.cn/domainevo.

In order to facilitate the use of researchers, we have developed a web server, where users can directly submit protein sequence files and select various parameters for protein evolution simulation. The system will send the results to the user's e-mail address. The graphical user interface of the website is shown in Figure 6.

### Input File

The user uploads a protein sequence file in FASTA format by clicking the button “Submit Sequence.”

### Ancestral Domain

The ancestral domains are the initial state of the cell in the middle of the CA space. The model uses this parameter as a common ancestor of the protein. Once this parameter is filled in, all evolved proteins will contain this domain.

### E-Value

The E-value is a parameter used in the HMMER software; it is the expected number of false positives (non-homologous sequences) that scored this well or better. The E-value is a measure of statistical significance. The lower the E-value, the more significant the hit. Changing the value of E-value will cause the same protein to compare different domain architectures, and the default value is “1e-37.”

### Evo-Num

The number of times of model simulation.

### Top-Num

The model analyzes automatically the evolved proteins and sends the top top-num frequency of the domain architectures to users.

After submitting the protein sequence file and parameters, the system will compare the uploaded protein sequences based on HMMER. By setting the value of the E-value, each protein will generate a homology domain information file. The domain of each protein is then extracted and sorted according to the position of the domain in the protein sequence, such that each protein can be represented as a multi-domain sequence.

Next, the sliding window processing is performed on each multi-domain sequence (the sliding window size is 2), the matrix of probability from back to front and the matrix of probability from front to back are obtained by counting the frequency of the domain in pairs.

In the evolution process of the CA, the next time state of the cell is obtained according to the CA evolution rule. When evolution is terminated, the system removes the domain ∅ and generates a complete multi-domain protein. The user can control the number of proteins that the system simulates by adjusting the size of Evo-num.

After the evolution of the protein, the system calculates the frequency of each domain architecture based on the multi-domain protein of the original file and the multi-domain protein obtained by the proposed simulation model, sorts the frequency from large to small, and saves it as two files in comma-separated values (CSV) format. Since there are many protein domain architectures obtained by simulation, users can adjust the value of Top-num to preserve the domain architectures with high frequency.

**Figure 7:**
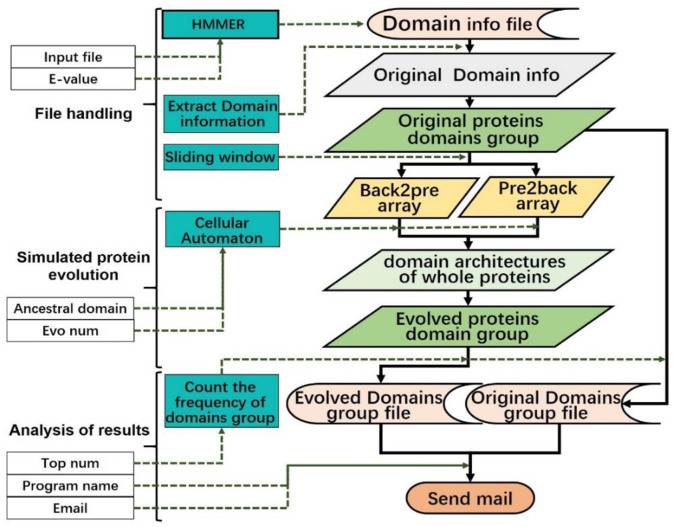
Data flow diagram of the model. Different types of data are represented by different background colors.

Finally, the system will send the results to the user's e-mail address. The contents of the e-mail are the parameters selected by the user, and in the attachment, there are two data files in CSV format. The CA model can be described by the flowchart in Figure 7.

## Simulating the Evolution of RhoGEF Domain in *Homo Sapiens* Proteins

### Materials

To evaluate the performance of the model, we used a number of multi-domain proteins associated with the conserved protein family “RhoGEF” to validate the validity of the model. The keyword “RhoGEF” was used to find the protein of the conserved protein family RhoGEF from the NCBI database. We selected the species *Homo sapiens* to download a multi-sequence file containing 1,597 proteins and used it as a protein sequence file P. In addition to the above methods, researchers can also build data sets by the following methods. The Conservative Domain Database (CDD, https://www.ncbi.nlm.nih.gov/cdd/) collects a large number of protein domains and domain families (Marchler-Bauer et al., 2016). Researchers can search for a conserved protein domain family in the CDD website and then click the “related protein” on the domain family description page to link to the NCBI website to download the related proteins.

### Model Performance Evaluation Under Various Parameters

After submitting the protein sequence file, we tested various parameters that may affect the evolution of protein simulation. The protein sequence file P is processed into a training data set P′ of the evolutionary simulation model:

(8) $$\text{P}=\left(\begin{array}{c} {{\text{ p}}_{1}}^{\prime }\\ {{\text{p}}_{2}}^{\prime }\\ \begin{array}{l} .\\ .\\ . \end{array}\\ {p_{1597}}^{\prime } \end{array}\right)=\left(\begin{array}{cccc} {{\text{d}}_{1,1}}^{\prime } & {{\text{d}}_{2,1}}^{\prime } & ... & {{\text{d}}_{1,w}}^{\prime }\\ {{\text{d}}_{2,1}}^{\prime } & {{\text{d}}_{2,2}}^{\prime } & ... & {{\text{d}}_{2,1}}^{\prime }\\ \begin{array}{l} .\\ .\\ . \end{array} & \begin{array}{l} .\\ .\\ . \end{array} & \begin{array}{l} .\\ .\\ . \end{array} & \begin{array}{l} .\\ .\\ . \end{array}\\ {p_{1597,1}}^{\prime } & {p_{1597,2}}^{\prime } & ... & {p_{1597,l}}^{\prime } \end{array}\right)$$

*P*^*evo*^ represents a multi-domain sequence file from the simulation of protein evolution in this model.

(9) $$\begin{array}{r} P^{evo}=\left(\begin{array}{c} D_{1}^{evo}\\ D_{2}^{evo}\\ \ldots \\ D_{Evo\text{\_}num}^{evo} \end{array}\right)=\left(\begin{array}{cccc} d_{1,1}^{evo} & d_{1,2}^{evo} & \cdots & d_{1,x}^{evo}\\ d_{2,1}^{evo} & d_{2,2}^{evo} & \cdots & d_{2,y}^{evo}\\ \vdots & \vdots & \vdots & \vdots \\ d_{Evo\text{\_}num,1}^{evo} & d_{Evo\text{\_}num,2}^{evo} & \cdots & d_{Evo\text{\_}num,\text{z}}^{evo} \end{array}\right) \end{array}$$

where $D_{n}^{evo}\left(1\leq n\leq Evo\text{\_}num\right)$ represents the simulation of the evolution of domain architectures of whole proteins (DAWPs), *x* is the number of domain in simulation protein $D_{1}^{evo}$, *y* is the number of domain in simulation protein $D_{2}^{evo}$, and so forth. Protein domains are represented by $d_{j,k}^{evo}$.

In order to examine the performance of a predictor in simulating domain evolution of proteins, *Hit*-*Acc* (DAWP), goodness of fit between the simulation proteins and nature protein in P′, used in this literature based on counting the type and number of the DAWPs. *Hit*-*Acc* (TAPD), goodness of fit between the triplet domain architectures in *P*′ and *P*^*evo*^, *Hit*-*Acc* (QAPD), goodness of fit between the quadruple domain architectures in *P*′ and *P*^*evo*^ as supplementary.

(10) $$\begin{array}{r} \text{Hit}–\text{Acc }\left(\text{DAWP}\right)=\frac{\sum_{i=1}^{Evo\text{\_}num}f^{evo}\left(D_{i}^{evo}\right)}{Evo\text{\_}num}\times 100 \end{array}$$

(11) $$f^{evo}\left(D_{i}^{evo}\right)=\left\{ \begin{array}{l} 1,if\;D_{i}^{evo}\;appeared\;in\;{\text{P}}^{\prime }\\ 0,otherwise \end{array}\right.$$

(12) $$\begin{array}{r} \text{Hit}–\text{Acc }\left(\text{TAPD}\right)=\frac{\sum_{i=1}^{m}\theta^{evo}\left(t_{i}\right)}{\sum_{i=1}^{k}\theta^{evo}\left(t_{i}^{evo}\right)}\times 100 \end{array}$$

(13) $$\left\{ \begin{array}{l} {\text{T}}^{\prime }=\left\{{t_{1}}^{\prime },{t_{2}}^{\prime },\ldots ,{t_{e}}^{\prime }\right\}\\ {\text{T}}^{\text{evo}}=\left\{t_{1}^{evo},t_{2}^{evo},\ldots ,t_{f}^{evo}\right\}\\ T={\text{T}}^{\prime }\cap {\text{T}}^{\text{evo}}=\left\{t_{1},t_{2},\ldots ,t_{r}\right\} \end{array}\right.$$

where T′ is the set of triplet domain architectures in P′, T^evo^ is the set of triplet domain architectures in P^evo^, ∩ represents the symbol for “intersection” in the set theory, $\theta^{evo}\left(t_{i}\right)$ represents the count hits of triplet domain architectures *t*_*i*_ in *P*^*evo*^, and $\theta^{evo}\left(t_{i}^{evo}\right)$ represents the hits of triplet domain architectures $t_{i}^{evo}$ in *P*^*evo*^.

(14) $$\begin{array}{r} \text{Hit}–\text{Acc }\left(\text{QAPD}\right)=\frac{\sum_{i=1}^{m}\varphi^{evo}\left(q_{i}\right)}{\sum_{i=1}^{k}\varphi^{evo}\left(q_{i}^{evo}\right)}\times 100 \end{array}$$

(15) $$\left\{ \begin{array}{l} {\text{Q}}^{\prime }=\left\{{q_{1}}^{\prime },{q_{2}}^{\prime },\ldots ,{q_{h}}^{\prime }\right\}\\ {\text{Q}}^{\text{evo}}=\left\{q_{1}^{evo},|q_{2}^{evo},\ldots ,q_{a}^{evo}\right\}\\ Q={\text{Q}}^{\prime }\cap {\text{Q}}^{\text{evo}}=\left\{q_{1},q_{2},\ldots ,q_{s}\right\} \end{array}\right.$$

where *Q*′ is the set of quadruple domain architectures in *P*′, Q^*evo*^ is the set of quadruple domain architectures in *P*^*evo*^, $\varphi^{evo}\left(q_{i}\right)$ represents the hits of quadruple domain architectures *q*_*i*_ in *P*^*evo*^, and $\varphi^{evo}\left(q_{i}^{evo}\right)$ represents the hits of quadruple domain architectures $q_{i}^{evo}$ appearing in *P*^*evo*^.

In the process of testing system performance, we used HMMER to perform multi-domain sequence alignment on *Homo sapiens*' protein family RhoGEF, and set the value of E-values from 1e-1 to 1e-50, so that the protein domain information file corresponding to E-values would be generated, and 50 simulation training data sets would be obtained after processing the file. The smaller the value of E-value, the higher the accuracy of the aligned homology domain. Changing the value of E-value will cause the same protein match different domain architectures when the value of E-value is 1e-1. A total of 228 domains existed in the simulation data set P, and the number of types of protein domain architectures was 326. When the value of E-value is 1e-37, there are 43 domains in the simulation data set P, the number of types of protein domain architecture is 55, and the most complex protein domain architecture is {′*I*-*set*′,′*V*-*set*′,′*Ig*′,′*Ig*′,′*Ig*′,′*Izumo*-*Ig*′,′*Ig*′,′*Ig*′,′*Pkinase*′,′*Pkinase*′}. The 10 most frequent protein domains are shown in Table 1.

**Table 1:** The 10 most frequent protein domains when E-values are 1e-1 and 1e-37, respectively.

**E-value : 1e-1**			**E-value : 1e-37**		
**Domain**	**Frequency**	**Probability (%)**	**Domain**	**Frequency**	**Probability (%)**
PH	2,776	20.07	RhoGEF	619	15.10
RhoGEF	1,440	10.41	Ig	243	5.93
SH3	1,091	7.89	PH	172	4.20
IQ	473	3.42	SH3	129	3.15
Ig	379	2.74	Pkinase	115	2.80
PDZ	314	2.27	RhoGEF67	73	1.78
C1	207	1.50	RGS-like	55	1.34
FYVE	204	1.47	RasGEF	52	1.27
EF-hand	190	1.37	I-set	49	1.20
Pkinase	187	1.35	V-set	48	1.17

**Figure 8:**
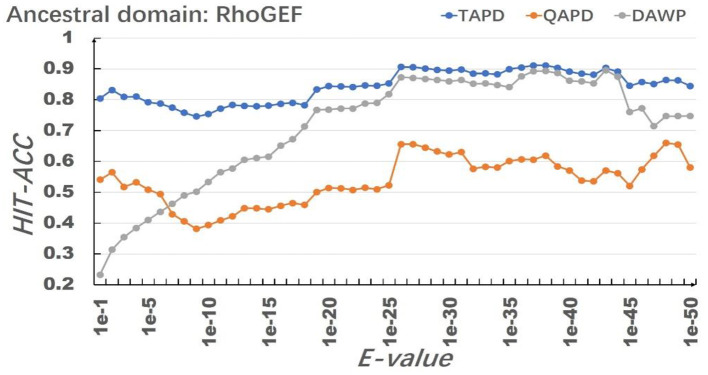
Results of the selection of RhoGEF as an ancestral protein domain.

**Figure 9:**
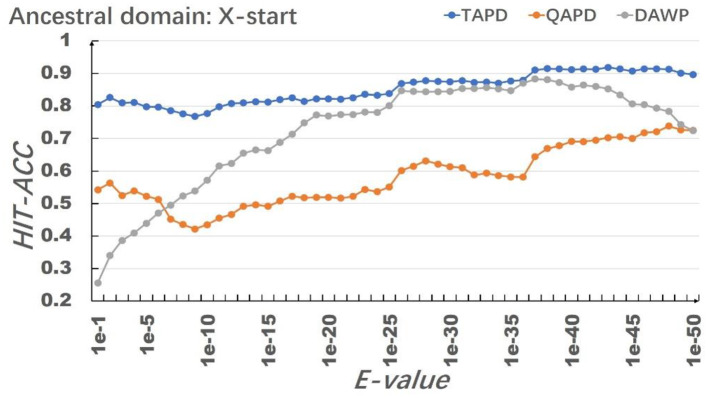
Results of the selection of *X*-start as an ancestral protein domain.

**Figure 10:**
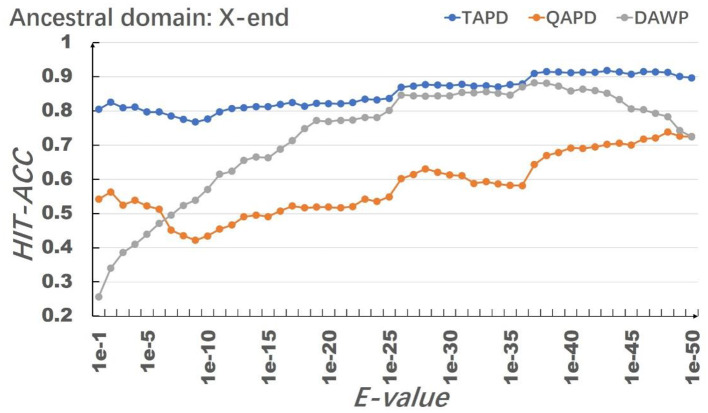
Results of the selection of *X*-end as an ancestral protein domain.

The model uses RhoGEF, *X*. -start, and *X*-end as ancestral protein domains to simulate each training set, and each training set simulates 50,000 proteins represented by multiple domain sequences. In order to eliminate the impact of individual results on the model evaluation, we repeated the simulation of each parameter combination 50 times and then calculated the average value of Hit-Acc. as the evaluation of the model; the obtned results are shown in Figures 8–10.

When the ancestral protein domain is RhoGEF, the Hit-Acc (TAPD). and Hit-Acc (DAWP) reach the maximum; when the E-value is 1e−43, the Hit-Acc (TAPD) is 90.27%, the Hit-Acc (DAWP) is 89.59%. When the ancestral protein domain is *X*-start or *X*-end, the test results are basically the same as the ancestral protein domain RhoGEF. The Hit-Acc (TAPD) and Hit-Acc (DAWP) reach the maximum when the E-value is 1e-37, the Hit-Acc (TAPD) is 91.01%, and the Hit-Acc (DAWP) is 88.26%. These test results show that the model has good simulation characteristics and robustness. The model can also get better results in most cases by using *X*-start and *X*-end as the ancestral protein domain for simulated evolution. The reason is that this model uses HMMER for automated annotation of protein domains. The smaller the E-values, the fewer domains are annotated. Some proteins will not annotate the ancestral domain because the set E-value is too small. For example, if the E-value is 1e-1, 1,440 proteins are annotated with the RhoGEF domain; if the E-value is 1e-37, only 619 proteins are annotated with the RhoGEF domain. In order to solve this problem, the model added *X*-start and *X*-end to the left and right ends of each protein when composing the protein simulation training data set. If the model uses *X*-start or *X*-end as the ancestral protein domain, each protein domain architecture could be simulated, which also allows the system to simulate more protein domain architectures that appear in the original file. This also means that if the user does not know the ancestor domain of the submitted protein sequence file, *X*-start or *X*-end can be used as the ancestral domain for protein simulation. It can be seen from the results that the model can effectively simulate the DAWPs under various parameters. It also simulated existing triplet and quadruplet domain architectures successfully. Analysis on the results shows that the model not only has good stability but also has strong robustness.

**Figure 11:**
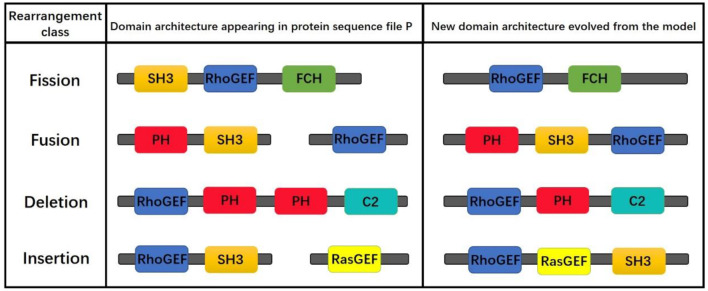
Examples of fission, fusion, deletion, and insertion in the evolution of the model.

Although the proposed model simulating the evolution of protein domain architectures only by fusion operation, the obtained results do contain the results of insertion, deletion, and mutation operations. For example, given that the simulation starts with “RhoGEF,” if one result is “RhoGEF-PH-PH-C2” and another is “RhoGEF-PH-C2,” then we can assume that the later one is the result of the fore in which the “PH” was deleted. The operations are shown in Figure 11.

Moreover, statistical analysis by the triplet and quadruple domain architectures derived from the evolution of the model shows that only a few domains contain a large number of immediate neighbors, and most of the domains contain only a small number of immediate neighbors, the frequency distribution of each domain neighbor in accordance with a power law (Qian et al., 2001). Although we used domain pair probability matrices to simulate the protein domain architecture, statistical results show that some triplets and quadruped domains are highly repetitive and appear in many sequences. In a statistical sense, these triplet and quadruple protein domain architectures are often over-expressed and highly abundant, which is consistent with the supra-domains concept (Vogel et al., 2004). This further illustrates the effectiveness of this model in simulating the evolution of protein domain architecture.

## Conclusions

The goal of this work was to provide a tool that can reveal how domain architectures have evolved in protein sequences. This tool is very important in analyzing orthologous relationships between proteins in different organisms. CA has been applied in many fundamental issues in biology. Some works have already been devoted to providing a framework for simulation evolution of protein and DNA sequences. In this work, 1D probabilistic CA is used to simulate the evolution of protein domain. This model simulates the fission, fusion, deletion, and insertion of the natural evolution processes by randomly appointing transitional rules. This is the reason that our model is more in line with the natural characteristics of protein evolution. Through this website, users can know the domain architecture distribution of the submitted protein sequence and can also view the evolution results of the model to predict the evolution direction of the protein sequence file with the emergence of new and frequent protein domain architecture.

## Data Availability Statement

Publicly available datasets were analyzed in this study. This data can be found here: access protein sequence data from NCBI, and searching HMM domains from Pfam with HMMER.

## Author Contributions

All authors listed have made a substantial, direct and intellectual contribution to the work, and approved it for publication.

## Conflict of Interest

The authors declare that the research was conducted in the absence of any commercial or financial relationships that could be construed as a potential conflict of interest.
